# Quantification of Glutamate and Aspartate by Ultra-High Performance Liquid Chromatography

**DOI:** 10.3390/molecules23061389

**Published:** 2018-06-08

**Authors:** Carlos Agius, Sabine von Tucher, Brigitte Poppenberger, Wilfried Rozhon

**Affiliations:** 1 Biotechnology of Horticultural Crops, TUM School of Life Sciences Weihenstephan, Technical University of Munich, Liesel-Beckmann-Straße 1, 85354 Freising, Germany; carlos.agius@tum.de (C.A.); brigitte.poppenberger@wzw.tum.de (B.P.); 2 Chair of Plant Nutrition, TUM School of Life Sciences Weihenstephan, Technical University of Munich, Emil-Ramann-Straße 2, 85350 Freising, Germany; tucher@wzw.tum.de

**Keywords:** aspartic acid, 2,4-dinitro-1-fluorobenzene, glutamic acid, ripening, tomato, ultra-high performance liquid chromatography

## Abstract

Glutamic and aspartic acid fulfil numerous functions in organisms. They are proteinogenic amino acids, they function as neurotransmitters, and glutamic acid links the citrate cycle with amino acid metabolism. In addition, glutamic acid is a precursor for many bioactive molecules like γ-aminobutyric acid (GABA). In tomatoes, glutamic acid accumulates in ripening fruits. Here we present a simple and rapid method for quantification of glutamate and aspartate in tomatoes. A cleared extract is prepared and 2-aminoadipic acid added as internal standard. Subsequently, the amino acids are derivatised with 2,4-dinitro-1-fluorobenzene under alkaline conditions. The derivatives are separated by ultra-high performance liquid chromatography using a phenyl-hexyl column and 50 mM *N*-methylmorpholine/acetate buffer pH 7.4 containing 12% acetonitrile as eluent and detected by UV absorption at 363 nm. The whole analysis time including separation and column equilibration takes less than 2.8 min with a flow rate of 1 mL/min and less than 1.6 min with a flow rate of 2 mL/min, making this method suitable for high-throughput applications. The method shows excellent reproducibility with intra- and inter-day SDs of approximately 4% for both aspartic and glutamic acid. Using this method we show that the glutamate/aspartate ratio changes significantly during fruit ripening.

## 1. Introduction

Glutamic acid and aspartic acid are the only proteinogenic amino acids with acidic side chains. Since their side chains are charged and hydrophilic they are frequently found on the surface of proteins. There they enhance the solubility of the protein and may also be involved in ionic protein–protein interactions. Due to their ability to act as proton donators and acceptors, glutamate and aspartate residues are also frequently found in active centres of enzymes [1], where they may be involved in the catalytic reaction and/or substrate binding. In addition to their role as building blocks of proteins, both amino acids fulfil a number of functions in their free form. Glutamic acid is a key compound in metabolism since it links the citrate cycle with amino acid metabolism [2]. Moreover, it is a precursor for a multitude of compounds including the amino acid glutamine and γ-aminobutyric acid (GABA), the chief inhibitory neurotransmitter in the mammalian nervous system [3]. Glutamate itself is the principal excitatory neurotransmitter in the mammalian brain [4], where it is involved in cognitive processes like learning and memory [5]. Aspartate can also stimulate neuronal receptors but less efficiently than glutamate [6,7]. In addition, glutamate is crucial for detoxification of ammonia in the mammalian brain [8] and for detoxification of xenobiotic compounds in many organisms [9,10]. In plants, glutamate has an essential role in amino acid anabolism. The α-amino group allows assimilation and dissimilation of ammonia and is the building block of all other amino acids. In plants GABA, arginine and proline are synthesised from glutamate [11,12]. Glutamate is also a precursor for synthesis of chlorophyll [13].

In a variety of vegetables and fruits such as tomatoes, green peas, mushrooms, and cabbage glutamic acid is found in considerable amounts. Ripe tomato fruits contain aspartate and glutamate in amounts of approximately 800 and 3000 mg/kg, respectively. During ripening of tomatoes, changes of aspartate and glutamate concentrations happen. In the case of glutamate, a red, fully ripened tomato contains a significantly higher concentration of glutamate than an immature green tomato. Aspartate is also reported to increase during tomato ripening, though at a much lower magnitude [14]. Amongst the total free amino acids in ripened tomato fruit, glutamate represents approximately 55% of the relative molar concentration, making it the main free amino acid [15].

Due to the importance of glutamate and aspartate in metabolism and development, a number of methods have been developed for their quantification. Both compounds can be quantified together with other amino acids by chromatographic methods. Classically, amino acids are separated in so-called amino acid analysers by cation exchange chromatography and post-column derivatisation with ninhydrin to give coloured compounds that are detected at 440 nm and 570 nm [16]. For more sensitive and rapid quantification a number of high-performance liquid chromatography (HPLC) techniques, mainly with pre-column derivatisation, were reported. A widely applied reagent is phenyl isothiocyanate [17,18,19], which is also part of Edman chemistry for protein sequencing [20]. For highly sensitive quantification of amino acids, derivatisation with o-phthaldialdehyde (OPA) and a thiole like 2-mercaptoethanol [21,22,23] or more recently 3-mercaptopropionic acid [24] and fluorescence detection is frequently applied. Disadvantages of o-phthaldialdehyde derivatisation are the low stability of the derivatives and that it does not react with proline, making a second derivatisation step with fluorenylmethyloxycarbonyl (FMOC) necessary [25]. In contrast, 6-aminoquinoline-*N*-hydroxy-succinimidyl carbamate (AQC) reacts rapidly with both primary and secondary amines [26] to highly fluorescent derivatives. Disadvantages of AQC are that the emission wavelength depends on the water content of the eluent, that in solutions with a high water content significant fluorescence quenching is observed [26], and that the reagent is very expensive. More recently, derivatisation with AQC has been applied for quantification of amino acids by liquid chromatography-mass spectrometry (LC-MS) [27] and liquid chromatography-tandem mass spectrometry (LC-MS/MS) in plant material [28]. In these methods the AQC tag is used to enhance binding of the amino acids to the reversed phase column and for improvement of detection. Underivatised amino acids can also be analysed by LC-MS/MS [29,30]. LC-MS/MS-based methods are often extremely sensitive and highly selective. However, major drawbacks are frequently observed matrix effects caused by ion suppression [31], which can be particularly significant for methods with limited separation of the amino acids from the matrix [30]. These effects can be compensated by using stable isotope labelled standards. However, such standards are very costly and also the equipment required for LC-MS/MS is very expensive and requires specially trained personnel. In addition, the high sensitivity offered by LC-MS/MS is not an advantage for analysis of amino acids in tomatoes since they contain free amino acids in the mg/L to g/L range, which makes several hundred-fold dilution necessary and may thereby even introduce dilution errors. In addition, for tomato breeding and fruit quality assessment only the glutamate and aspartate contents are relevant since these two amino acids have, in contrast to other amino acids, a significant impact on the taste [32,33].

Underivatised amino acids can also be detected electrochemically [34,35,36]. Nevertheless, amino acids are often derivatised to improve sensitivity or enhance separation on reverse phase columns [37,38]. Although electrochemical detection of amino acids offers high sensitivity this technique is comparatively little applied since the use of electrochemical detectors is not very widespread.

For specific quantification of glutamate enzyme assays are available where glutamate is oxidised by glutamate oxidase to α-ketoglutarate and hydrogen peroxide, the latter reacts subsequently by catalysis of horse radish peroxidase with an artificial substrate to a fluorescent compound [39]. Alternatively, glutamate dehydrogenase can be used to reduce NAD (oxidised β-nicotinamide adenine dinucleotide) to NADH (reduced β-nicotinamide adenine dinucleotide), which can be either directly measured at 340 nm [40] or used for reduction of a formazan to a blue dye [41]. Similar enzymatic assays have also been developed for quantification of aspartic acid [42,43]. Enzymatic assays for both glutamate and aspartate have been commercialised as test kits allowing convenient quantification of these metabolites. However, disadvantages are the limited shelf-life of the reagents, sensitivity to inhibitors, and the relatively high costs per assay.

**Figure 1:**
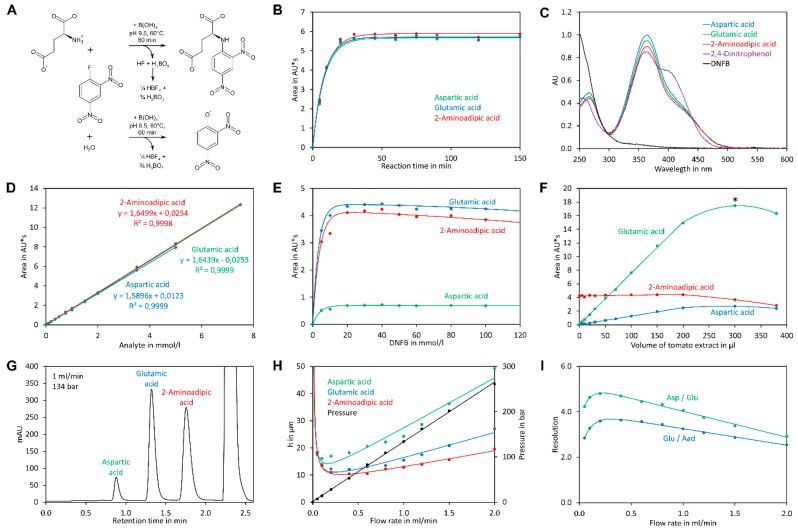
Optimisation of derivatisation and separation. (**A**) Derivatisation of glutamic acid with 2,4-dinitrofluorobenzene (DNFB); (**B**) Reaction kinetic of derivatisation of aspartic, glutamic and 2-aminoadipic acid with 30 mM DNFB in borate buffer pH 9.5 at 60 °C; (**C**) UV spectra of the 2,4-dinitrophenyl derivatives measured in elution buffer A; (**D**) Detector response of the three amino acids at 363 nm; (**E**) The impact of different DNFB concentrations on the derivatisation reaction; (**F**) The effect of different sample volumes on derivatisation. The sample marked with an asterisk exceeded the upper limit of the detector (2 AU) and the area is therefore underestimated; (**G**) A typical UHPLC chromatogram of a tomato sample; (**H**) van Deemter diagram for the derivatised amino acids. The pressure is shown on the secondary axis; (**I**) Dependency of resolution on the flow rate.

Here we present a quick and high-throughput ultra-high performance liquid chromatography (UHPLC) method for simultaneous quantification of glutamate and aspartate in tomato fruit by pre-column derivatisation with 2,4-dinitro-1-fluorobenzene (DNFB) and UV detection at 363 nm. DNFB was originally introduced by Frederick Sanger for labelling the N-terminal amino acid of proteins and peptides [44]. The derivatisation of amino acids with DNFB is unique since an amine bond is formed (Figure 1A), which can even resist harsh conditions required for hydrolysis of proteins (incubation in 6 M hydrochloric acid at 110 °C for 24 h). In addition, the amine nitrogen in the obtained derivative has, due to the electron capturing properties of the 2,4-dinitrobenzene moiety, an extremely low nucleophilicity and thus double derivatisation is not observed. DNFB has been used for labelling of free amino acids and subsequent quantification of the derivatives by HPLC with UV detection, but the formation of 2,4-dinitrophenol from the reaction of excess reagent with water (Figure 1A) made this method complicated [45]. Recently, a method for quantification of glutamate by pre-column derivatisation with 2,4-dinitrofluorobenzene and subsequent reversed-phase HPLC has been described. However, this method required removal of 2,4-dinitrophenol by extraction with diethyl ether, making sample preparation tedious [46].

Here we provided an optimised protocol for derivatisation and chromatographic conditions that make removal of 2,4-dinitrophenol unnecessary and allow separation including column re-equilibration in 1.6 min. Importantly, the derivatisation reagent and the required buffers are inexpensive. Thus, the method is simple, rapid, and cost-efficient.

## 2. Results and Discussion

### 2.1. Method Development

**Figure A1:**
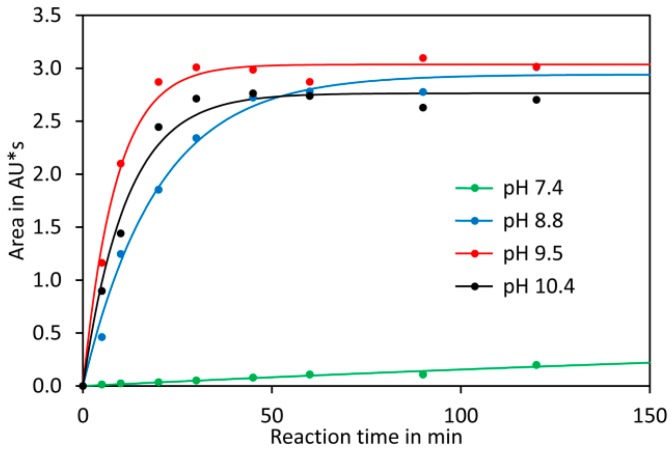
Kinetics of derivatisation at different pH values. Aspartic acid was derivatised with 30 mM DNFB in *N*-methylmorpholine/acetic acid buffer pH 7.4, sodium hydrogen carbonate/sodium carbonate buffer pH 8.8, sodium borate buffer pH 9.5, and triethylamine/acetic acid buffer pH 10.4 at 60 °C. The reactions were stopped at the indicated time points by addition of acetic acid and the samples were analysed by UHPLC.

Derivatisation of amino acids with DNFB requires a moderate basic pH since the amino group must be present in its deprotonated form to be sufficiently nucleophilic to substitute the fluoro residue of the reagent (Figure 1A). To optimise the conditions for derivatisation, aspartic acid was incubated in different buffers with DNFB at 60 °C (Appendix A, Figure A1). At pH 7.4 the reaction proceeded extremely slowly, while it was finished in less than 100 min at pH 8.8, and in less than 40 min at pH 9.5 and 10.4. The best result with respect to peak areas was obtained with borate buffer pH 9.5. Borate buffer has the additional advantage that it rapidly forms tetrafluoroborate with fluoride (Figure 1A), a by-product of the derivatisation reaction with DNFB that may otherwise damage silica-based columns by solubilising the column matrix as hexafluorosilicic acid. Because of this benefit and since the best signals were obtained with borate buffer, it was used for all subsequent experiments.

**Figure 2:**
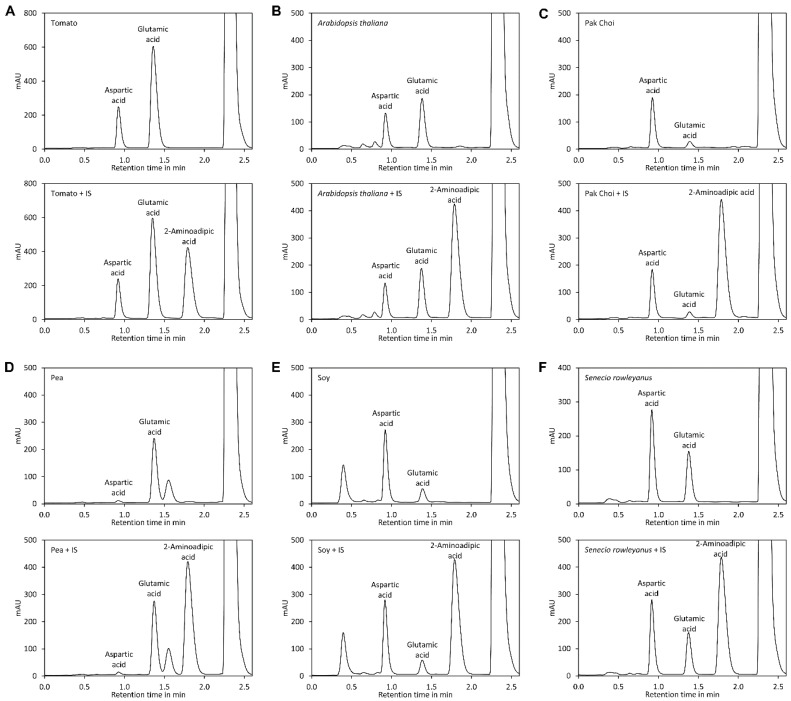
2-Aminoadipic acid is not present in plant samples. (**A**) Chromatogram of a tomato extract (5 µL tomato extract were derivatised in a reaction with a total volume of 120 µL) derivatised with DNFB and analysed by UHPLC with a flow rate of 1 mL/min. The sample in the upper panel was not spiked, the sample in the lower panel was spiked with 10 µL of 2-aminoadipic acid 1000 mg/L. All other samples were treated in the same way and analysed without spike (upper panels) and spiked with 10 µL of 2-aminoadipic acid 1000 mg/L (lower panels); (**B**) Sap of *Arabidopsis thaliana* leaves (30 µL); (**C**) Sap of pak choi petioles (30 µL); (**D**) Sap of unripe peas (2 µL); (**E**) Sap of soy leaves (20 µL); (**F**) Sap of *Senecio rowleaynus* leaves (30 µL).

**Figure A2:**
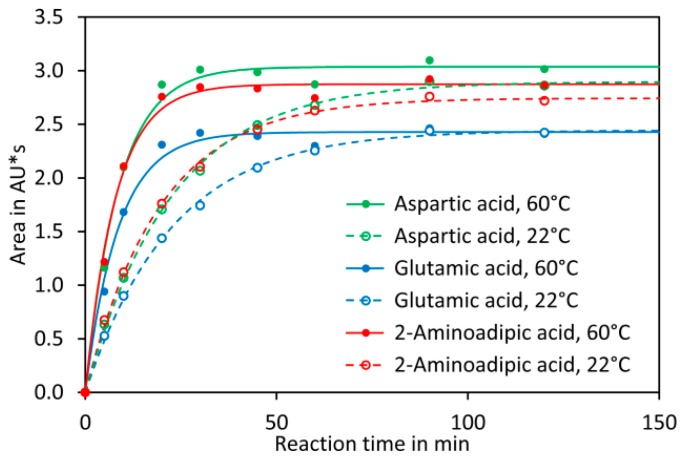
Kinetics of derivatisation at 60 °C and 22 °C. A mixture containing aspartic, glutamic, and 2-aminoadipic acid was derivatised with 30 mM DNFB in sodium borate buffer pH 9.5 at 60 °C and room temperature (22 °C). The reactions were stopped at the indicated time points and the samples analysed by UHPLC.

Reaction of DNFB with aspartic acid, glutamic acid, and 2-aminoadipic acid, which was later used as internal standard, showed that the reaction proceeded with a similar kinetic (Figure 1B) and that the obtained derivatives showed similar UV spectra with the absorption maxima at 363 nm (Figure 1C). Also 2,4-dinitrophenol formed by reaction of excess reagent with water has an absorption maximum at 363 nm. The derivatisation reaction proceeded more rapidly at 60 °C, where it finished in less than 40 min, than at room temperature, where it finished after approximately 2 h (Appendix A, Figure A2). For the further experiments we used 60 °C and a reaction time of 1 h, although it is also possible to derivatise the amino acids at room temperature for at least 2 h. Subsequently, the reaction was stopped by addition of acetic acid. Acidification of the reaction is also necessary to obtain sharper peaks since aspartic acid, in particular, tends to give broad and sometimes even doubled peaks if the basic reaction mixture is directly injected (data not shown). Aspartic acid, glutamic acid, and 2-aminoadipic acid showed perfectly linear signals and almost identical molar detector response factors as indicated by nearly identical slopes of the calibration curves (Figure 1D). The reason for this observation is that only the introduced chromophore, the 2,4-dinitrophenyl group, absorbs strongly at 363 nm while the amino acids residues do not absorb at that wavelength. Consequently, the derivatives of aspartic acid, glutamic acid, and the internal standard 2-aminoadipic acid have almost identical molar extinction coefficients at 363 nm. In principle, this allows for determination of the molar aspartate to glutamate ratio in samples even without establishing a calibration curve since the ratio of the peak areas of aspartate to glutamate is equal to their molar ratio. However, before such an assay is performed, it is essential to confirm that identical detector responses for aspartate and glutamate are obtained with the used chromatographic system since, besides the extinction coefficient, the peak shape also has an impact on the peak area. The almost identical detector response of 2-aminoadipic acid and its absence from plant samples including tomato fruits, *Arabidopsis thaliana* leaves, pak choi petioles, peas, and soy leaves (Figure 2) makes this compound an optimal internal standard.

To investigate the optimal concentration of reagent a tomato extract was prepared and derivatised with different concentrations of DNFB. We decided to use a real sample for this experiment because tomato, in addition to glutamate and aspartate, may contain significant amounts of compounds reacting with the reagent. The tomato extract was spiked with 2-aminoadipic acid. The best signals were obtained with a DNFB concentration of 20 mM to 50 mM (Figure 1E). At higher concentrations a precipitate was formed and the signal declined slightly, which may be explained by acidification of the reaction since reaction of DNFB with amino acids and water forms an equimolar amount of acid (Figure 1A). For further experiments 30 mM DNFB was used. To investigate the impact of the sample amount, different volumes of tomato extract were spiked with 2-aminoadipic acid. For each reaction the same amount of 2-aminoadipic acid was added irrespective of the volume of tomato extract used. A linear increase of the signals of aspartic acid and glutamic acid was observed up to 200 µL sample. Importantly, the signal of added 2-aminoadipic acids remained constant in these samples (Figure 1F). Addition of more sample led to a decline of the signal. In the case of glutamate this can be explained in part by the fact that the obtained peak exceeded the upper limit of the detector. However, the decline is likely also the result of acidification of the reaction by organic acids, mainly citric acid, present in tomato fruits at a considerable concentration [47,48,49,50].

**Figure 3:**
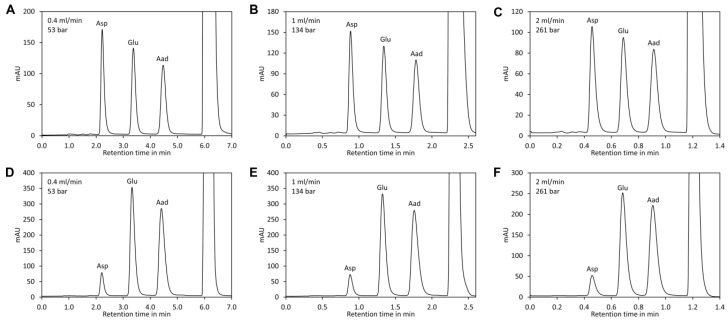
Impact of the flow rate on separation. A sample of standard containing equimolar amounts of aspartic acid (Asp), glutamic acid (Glu), and 2-aminoadipic acid (Aad) was derivatised with DNFB and analysed by UHPLC with a flow rate of (**A**) 0.4 mL/min, (**B**) 1 mL/min and (**C**) 2 mL/min. The injection volume was 5 µL. A tomato sample was derivatised with DNFB and analysed under the same conditions: (**D**) 0.4 mL/min, (**E**) 1 mL/min, and (**F**) 2 mL/min. The injection volume was 2 µL.

For separation of DNB-derivatised amino acids the presence of huge amounts of 2,4-dinitrophenol is challenging and measures for removal of this by-product are required [45,46]. However, we observed that aspartic acid, glutamic acid, and 2-aminoadipic acid derivatised with DNFB elute much faster than 2,4-dinitrophenol if neutral to slightly basic eluents are used, rendering removal of 2,4-dinitrophenol unnecessary. Under these conditions, glutamic acid and aspartic acid are also well separated from the other amino acids. In neutral to slightly alkaline conditions, the DNFB derivatives of glutamic acid and aspartic acid have two negative charges, while the DNFB derivatives of all other proteinogenic amino acids and the by-product 2,4-dinitrophenol have a single negative charge. Amines and ammonia form neutral derivatives. Thus, derivatised glutamic acid and aspartic acid are significantly less retarded than the other derivatives and the by-product 2,4-dinitrophenol and appear first in the chromatogram. However, it is important to mention that this selectivity is lost under acidic conditions, where glutamic acid and aspartic acid appear together with the other polar amino acids. We found that a buffer consisting of 50 mM *N*-methylmorpholine set with acetic acid to pH 7.4 and containing 12% acetonitrile as organic modifier is ideal since it allows excellent isocratic separation of the three derivatised amino acids (Figure 1G). In addition, it has a high buffering capacity and is fully miscible with acetonitrile in any ratio. After the isocratic step a short pulse with a high concentration of acetonitrile efficiently removes derivatives of other amino acids, reagent, and the by-product 2,4-dinitrophenol from the column. Separation was optimal with phenyl-hexyl columns since the peaks of aspartate, glutamate, and the internal standard 2-aminoadipic acid were equally distributed. In contrast, C18 columns retained derivatised 2-aminoadipic acid much more strongly than the two other amino acids, which prolonged the time required for optimal separation. To investigate the impact of the flow rate on separation, a van Deemter plot [51] was recorded (Figure 1H). For convenience, the flow rate (mL/min) rather than the velocity (m/s) of the mobile phase was plotted on the x-axis of the diagram. The diagram indicated that optimal separation is achieved at a flow rate of approximately 0.4 mL/min. However, separation was very good at a flow rate of 1 mL/min and even at 2 mL/min good resolution was obtained (Figure 1I and Figure 3). At a flow rate of 1 mL/min the whole separation including re-equilibration of the column required only 2.6 min. Since the system required 10 s for synchronisation of the system controller, autosampler, and UV detector, the time required for one sample was in total less than 2.8 min. At a flow rate of 2 mL/min the total time required for analysis of one sample was less than 1.6 min. Thus, the method is suitable for high-throughput applications.

To assess reproducibility of the method a tomato extract was obtained from both red ripe and green unripe *Solanum lycopersicum* cv. Gustafson cherry tomatoes and analysed in quintuplicate on five consecutive days. The results for red ripe tomatoes on individual days (intra-day) ranged from 3765 mg/L to 4011 mg/L for glutamate with relative standard deviations (RSDs) from 1.9% to 3.8%. Aspartate was in the range of 729 mg/L to 770 mg/L with RSDs ranging from 1.8% to 4.6%. The overall (inter-day) results for glutamate and aspartate were 3885 mg/L (RSD: 2.6%) and 747 mg/L (RSD: 2.5%), respectively, for red ripe tomatoes (Table 1). For green tomato fruits values for glutamate of 428 mg/L to 492 mg/L with RSDs of 1.3% to 6.8% and for aspartic acid ranging from 587 mg/L to 653 mg/L with RSDs of 2.5% to 8.3% were obtained. The inter-day results for glutamate and aspartate were 454 mg/L (RSD: 5.5%) and 602 mg/L (RSD 4.9%) (Table 2). These values are similar to a recent study with LC-MS [27], where an average inter-day reproducibility of approximately 4% for both glutamate and aspartate was reported, and better than a study with UHPLC high-resolution quadrupole orbitrap mass spectrometry [30], where an inter-day reproducibility of 20% for glutamate and 25% for aspartate was reported.

In summary, the method combines a simple derivatisation procedure with rapid separation and good reproducibility.

### 2.2. Comparison of Glutamate Quantification by UHPLC and Enzymatic Assay

**Figure 4:**
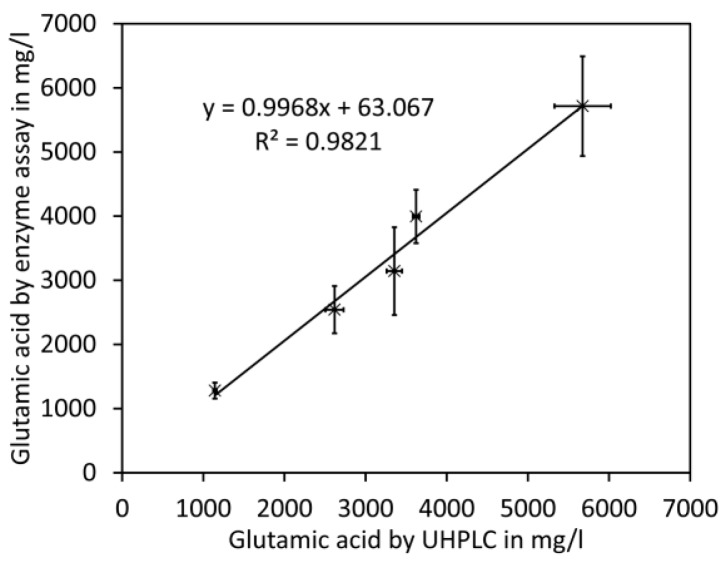
Comparison with enzymatic assay for glutamate. Different cultivars of cherry tomato fruits were analysed by UHPLC and enzyme test. The data shown are average values and error bars representing SD of 2 to 4 (enzyme test) or 3 (UHPLC) independent measurements (see Appendix A, Table A1 for details).

For the comparison of glutamate analyses by UHPLC and enzymatic assay, commercial cherry tomato fruits (*Solanum lycopersicum*) of five different cultivars purchased in a supermarket in July 2017 were used. Tomato fruit homogenates were prepared, subdivided, and processed for UHPLC or the enzyme test. A close linear relationship was obtained between both methods with a slope close to 1.0 (Figure 4 and Appendix A, Table A1). However, standard deviations for UHPLC were considerably lower compared to those for the enzyme test.

### 2.3. Application: Glutamate and Aspartate Content During Ripening of Tomato Fruit

**Figure 5:**
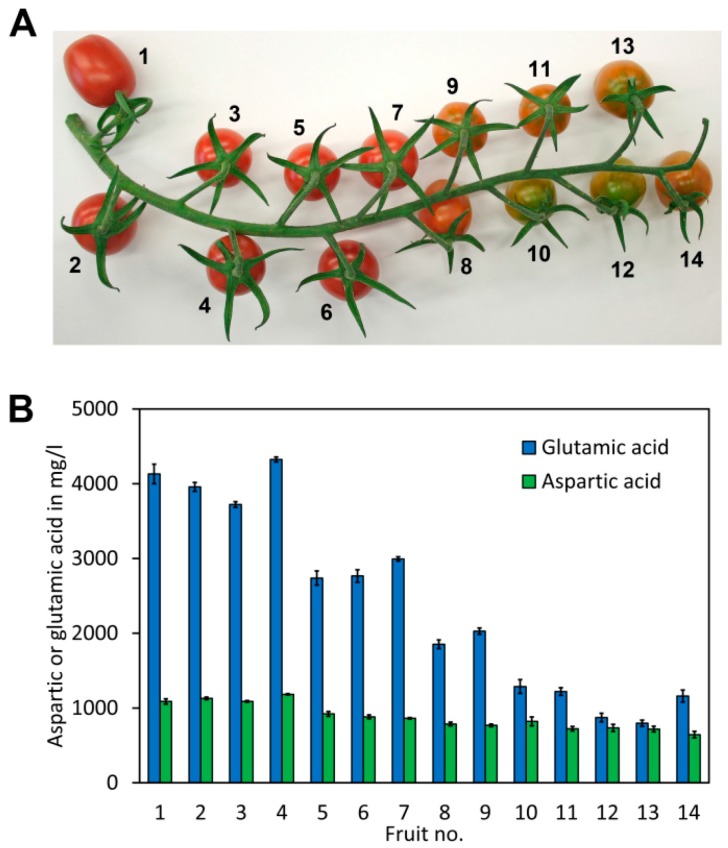
Analysis of glutamate and aspartate in different ripening stages of cherry tomato. (**A**) A cherry tomato truss (*Solanum lycopersicum* cv. Gustafson) with tomato fruits with different stages of ripeness. (**B**) Aspartate and glutamate content of the different cherry tomato fruits shown above. The columns and bars represent the average and SD of 4 independent measurements, respectively (see Appendix A, Table A2 for details).

To assess fluctuations in the glutamate and aspartate content of tomato fruit during ripening, a truss of cherry tomatoes (*S. lycopersicum* cv. Gustafson) exhibiting different levels of fruit ripening was used (Figure 5A). The fruits were numbered from 1 to 14, with 1 being the upper most fruit from the truss, presumably with the highest degree of ripeness due to earlier fruit set, and 14 being the last tomato fruit from the truss, with a lower degree of ripeness due to later fruit set. An extract was obtained from each fruit, derivatised and analysed in quadruplicate for glutamate and aspartate. The results showed a striking increase of the glutamate content during ripening (Figure 5B). An approximately 5-fold increase of the glutamate content was noted from the least ripe to the ripest fruits. Also, a significant increase in the aspartate content was noted in the riper fruits. However, the difference was, with 1.8-fold, clearly less pronounced than the difference in the glutamate content. 

The increase of the glutamate and aspartate content during ripening is believed to improve the taste of tomato fruit [32]. Similarly, the ratio of aspartate to glutamate in tomato fruit was also reported to impact the taste. A 4-fold higher concentration of glutamate with respect to aspartate based on weight in a synthetic tomato extract was able to reproduce a very similar taste to natural tomatoes [33]. This is in line with our work where the ratio of glutamate to aspartate increases from a ratio of approximately 1.1 to 3.8 during ripening (Appendix A, Table A2).

## 3. Materials and Methods

### 3.1. Sample Preparation

Tomato fruits were homogenised using an Ultra-Turrax disperser, the obtained homogenate was centrifuged at 4000 rpm for 5 min, and filtered through a syringe filter with a 0.2 µm nylon membrane. An aliquot of the filtered tomato sample (usually 50 µL) was transferred into an autosampler vial and spiked with 100 µL of l-2-aminoadipic acid 1600 mg/L as internal standard and water added to a total volume of 380 µL. For derivatisation, 320 µL 500 mM sodium borate buffer pH 9.5 and 400 µL 30 mM DNFB in acetonitrile (ACN) were added. The vials were closed and incubated at 60 °C for 1 h. Subsequently, the reaction was stopped by addition of 20 µL of glacial acetic and the samples were directly used for UHPLC. A detailed step-by-step protocol is included in Appendix B.

### 3.2. UHPLC

The liquid chromatography system consisted of a Shimadzu SCL-10Avp system controller, two Shimadzu LC-10ADvp pumps each equipped with a DGU-14A degasser and a FCV-10AL low pressure valve for eluent selection and connected for high pressure gradient elution, a SIL-10A autosampler equipped with a 50 µL sample loop, a CTO-10Avp column oven set to 25 °C, and a SPD-10A UV detector set to 363 nm. A Zorbax Eclipse 1.8 µm Phenyl-Hexyl 30 × 4.6 mm column (Agilent, Santa Clara, CA, USA) preceded by a SecurityGuard Phenyl-Hexyl 4 × 3 mm (Phenomenex, Torrance, CA, USA) precolumn was used for separation. For elution, a flow rate of 1 mL/min for separation in less than 2.8 min or of 2 mL/min for separation in less than 1.6 min was used. Eluent A consisted of 50 mM *N*-methylmorpholine adjusted with acetic acid to pH 7.4 in 12% acetonitrile and eluent B was 100% acetonitrile. Elution programs are listed in Appendix B, Table A4 and Table A5. Typically, an injection volume of 2 to 10 µL was used. Chromatograms were evaluated with the Clarity software package (DataApex, Prague, Czech Republic).

### 3.3. Enzyme Assay

Glutamate was quantified with a l-glutamic acid enzyme test kit (Cat. No. 10 139 092 035; R-Biopharm, Darmstadt, Germany) as recommended by the manufacturer. The assay is based on the formation of formazan (measured at 492 nm in a Lambda 20 spectrophotometer, Perkin Elmer, Norwalk, CA, USA) after the reduction of iodonitrotetrazolium chloride with NADH, which is derived from the oxidation of glutamic acid to 2-oxoglutarate. A calibration with glutamic acid was used for calculation of the glutamic acid concentration. For sample preparation, 1.0 g of the tomato fruit homogenate was diluted with 25 mL distilled water and heated to 70 °C for 30 min. After cooling, samples were adjusted to pH 8.0 with 0.1 M NaOH and filled up with distilled water to 50 mL. Aliquots of 100 or 200 µL were used for the test and analysed in triplicate.

## 4. Conclusions

Derivatisation with DNFB (Sanger’s reagent) and subsequent analysis by UHPLC is a simple, accurate, and cost-efficient method for analysis of glutamate and aspartate in tomato samples. Derivatisation with DNFB has the advantage that highly stable derivatives are formed. In addition, our optimised UHPLC separation makes sample clean-up, which was a main disadvantage of pervious methods using DNFB, unnecessary. The method shows excellent reproducibility with intra- and inter-day standard deviations of approximately 4% for both aspartic and glutamic acid. Because of simple sample preparation and fast separation, the method is highly suitable for high-throughput applications. In addition, since the maximal pressure remains below 300 bar, conventional high-pressure gradient HPLC equipment is sufficient for application of the method. We show that the method is suitable for quantification of glutamate and aspartate in tomato during fruit ripening. In addition, the method is also suitable for analysis of other plant materials.

## Figures and Tables

**Table 1:** Intra- and inter-day repeatability; sample of red, fully ripe tomato.

Experiment ^a,b^	Replicates	Aspartic Acid			Glutamic Acid		
		Average mg/L	SD mg/L	Rel. SD %	Average mg/L	SD mg/L	Rel. SD %
Day 1	5	733	34	4.6	3826	146	3.8
Day 2	5	763	4	1.8	3957	80	2.0
Day 3	5	770	7	2.3	4011	94	2.4
Day 4	5	740	21	2.9	3867	72	1.9
Day 5	5	729	27	3.6	3765	125	3.3
Inter-day	25	747	19	2.5	3885	99	2.6

^a^ Used variety: *Solanum lycopersicum* cv. Gustafson. ^b^ A flow rate of 1 mL/min was used for analysis.

**Table 2:** Intra- and inter-day repeatability; sample of green, unripe tomato.

Experiment ^a,b^	Replicates	Aspartic Acid			Glutamic Acid		
		Average mg/L	SD mg/L	Rel. SD %	Average mg/L	SD mg/L	Rel. SD %
Day 1	5	579	28	4.8	435	18	4.2
Day 2	5	587	14	2.5	428	9	2.2
Day 3	5	653	54	8.3	492	34	6.8
Day 4	5	598	31	5.2	458	26	5.7
Day 5	5	592	19	3.3	455	6	1.3
Inter-day	25	607	29	4.5	454	25	5.5

^a^ Used variety: *Solanum lycopersicum* cv. Gustafson. ^b^ A flow rate of 1 mL/min was used for analysis.

## Appendix A

**Table A1:** Analysis of glutamate in different tomato cultivars by UHPLC and an enzymatic test.

	UHPLC				Enzyme			
Experiment ^a^	Repeats	Average mg/L	SD mg/L	SD %	Repeats	Average mg/L	SD mg/L	SD %
Annamay, vine tomato, round, DE	3	3355	94	2.8	3	3143	683	21.7
Azura, cherry tomato, round, MA	3	1143	22	1.9	4	1280	124	9.7
Azura, cherry tomato, oval, MA	3	2617	112	4.3	3	2541	369	14.5
Mini Roma, vine tomato, oval, NL	3	3622	40	1.1	3	3995	417	10.4
Rivolo, cherry tomato, round, NL	3	5675	347	6.1	2	5715	777	13.6

^a^ DE: Germany; MA: Morocco; NL: Netherlands.

**Table A2:** Aspartate and glutamate contents during ripening of tomato fruits.

Fruit No. ^a,b^	Aspartic Acid		Glutamic Acid		Glu/Asp Ratio
	Average ^c^; mg/L	SD ^c^; mg/L	Average ^c^; mg/L	SD ^c^; mg/L	
1	1089	34	4131	132	3.8
2	1129	17	3958	59	3.5
3	1090	11	3722	38	3.4
4	1184	8	4325	33	3.7
5	922	32	2738	96	3.0
6	882	26	2766	82	3.1
7	863	9	2995	29	3.5
8	787	25	1853	58	2.4
9	769	18	2029	43	2.6
10	821	59	1288	93	1.6
11	723	30	1221	51	1.7
12	735	47	873	57	1.2
13	719	38	798	42	1.1
14	645	45	1160	81	1.8

^a^ The glutamate and aspartate contents of the fruits on the truss shown in Figure 5A were measured. ^b^ A flow rate of 1 mL/min was used for analysis. ^c^ The average and SD was calculated from 4 independent replicates.

## Appendix B. Step-by-Step Protocol for Quantification of Glutamic Acid and Aspartic Acid

### Appendix B.1. Reagents

Glu and Asp stock: glutamic acid 4000 mg/L, aspartic acid 1000 mg/L (transfer 508.8 mg monosodium glutamate monohydrate and 100.0 mg aspartic acid quantitatively into a 100 mL volumetric flask, add approximately 80 mL water, 10 mL ACN 100%, and 300 µL sodium hydroxide 4 mol/L and shake until the solid has completely dissolved. Add water to the mark. The solution is stable for at least 3 month at 4 °C and for at least 3 years at −20 °C)

Internal standard: l-2-aminoadipic acid 1.6 g/L (transfer 160.0 mg of 2-aminoadipic acid quantitatively into a 100 mL volumetric flask, add approximately 80 mL water, 10 mL ACN, and 400 µL sodium hydroxide 4 mol/L and shake until the solid has completely dissolved. Add water to the mark. The solution is stable for at least 3 month at 4 °C and for at least 3 years at −20 °C)

Derivatisation reagent: DNFB 30 mmol/L (mix 377 µL 2,4-dinitro-1-fluorobenzene with 100 mL ACN 100%. The solution can be kept in the dark at room temperature for at least 3 month)

Borate buffer 500 mM, pH 9.5 (dissolve 15.46 g boric acid in approximately 400 mL water and adjusted to pH 9.5 with sodium hydroxide 4 mol/L; add water to 500 mL)

Glacial acetic acid, 98–100%

Sodium hydroxide 4 mol/L (dissolve 16.0 g sodium hydroxide in water to a total volume of 100 mL)

Eluent A: 50 mM *N*-methylmorpholine set with acetic acid to pH 7.4 in 12% ACN (dissolve 10.12 g *N*-methylmorpholine in approximately 1600 mL water, add 186.4 g (corresponding to 240 mL) ACN 100%, and set the pH of the solution to 7.4 using glacial acetic acid. Transfer the solution quantitatively into a 2000 mL volumetric flask, add water to the mark and mix well. Filter the solution through a 0.22 µm nylon filter)

Eluent B: ACN 100%

### Appendix B.2. Sample Preparation

(1) Homogenise approximately 100 g tomato fruits with an Ultra-Turrax or a Warring Blender homogeniser.
(2) Transfer the homogenate into 50 mL tubes and centrifuge at 4000× *g* for 5 min.
(3) Filter approximately 2 mL of the supernatant through a 0.22 µm membrane syringe filter (PP, nylon and hydrophilic PTFE membranes are suitable) and use the filtrate for derivatisation. Note: the filtrate can be stored at −20° until analysis.
(4) Using a volumetric pipet transfer exactly 25 mL of the clear supernatant into a beaker placed on an analytical balance and weigh the supernatant. Calculate the density of the supernatant by dividing the weight by the volume. Note: the density is required for conversion of the concentration of aspartic acid and glutamic acid from g/l to the content in g/kg. However, if only the concentration (g/L) shall be calculated this step can be omitted.

### Appendix B.3. Derivatisation

(1) Transfer a suitable volume (usually 20 to 50 µL) of tomato extract into an autosampler vial and add water to a final volume of 380 µL. Note: if the sample amount is limiting it is possible to scale down all volumes indicated in step 1 to 6 by the factor 10.
(2) Add 100 µL internal standard and 320 µL borate buffer.
(3) Prepare standards in autosampler vials as indicated in Table A3.
(4) Add 400 µL derivatisation reagent to each standard and sample, close the vial, and shake vigorously.
(5) Incubate the samples at 60 °C for 1 h.
(6) Add 20 µL of glacial acetic acid to each sample, close the vial, and shake vigorously.
(7) The derivatised samples are analysed directly by UHPLC. It is also possible to store them in the dark at room temperature for up to one week prior to analysis.

**Table A3:** Preparation of standards.

No.	Glu and Asp Stock µL	Internal Standard µL	Water µL	Borate Buffer 500 mM, pH 9.5; µL
St 1	0	100	380	320
St 2	5	100	375	320
St 3	10	100	370	320
St 4	15	100	365	320
St 5	20	100	360	320
St 6	30	100	350	320
St 7	40	100	340	320
St 8	50	100	330	320
St 9	60	100	320	320
St 10	80	100	300	320
St 11	100	100	280	320

### Appendix B.4. UHPLC

Analyse the samples by UHPLC using the following conditions. An UHPLC system with a high pressure gradient and a minimal dead volume (less than 300 µL including the sample loop) must be used to allow application of the gradient indicated below. Note: the system used in this study had a dead volume of 200 µL. Use the following settings:

Column: Zorbax Eclipse 1.8 µm Phenyl-Hexyl 30 × 4.6 mm
Precolumn: SecurityGuard Phenyl-Hexyl 4 × 3 mm
Sample loop: 50 µL
Injection volume: 5 µL
Column oven: 25 °C
Detector: UV, 363 nm
Eluent A: 50 mM *N*-methylmorpholine set with acetic acid to pH 7.4 in 12% ACN
Eluent B: ACN 100%

Elution program: see Table A4 for an elution with a flow rate of 1 ml/min or Table A5 for elution with a flow rate of 2 mL/min.

**Table A4:** Elution program for 1 mL/min.

Time in min	Eluent A in%	Eluent B in %
0.0	100	0
1.8	100	0
1.9	20	80
2.1	20	80
2.2	100	0
2.6 (Stop) ^a^	100	0

^a^ The next sample is injected approximately 10 s later, thus the total analysis takes less than 2.8 min.

**Table A5:** Elution program for 2 mL/min.

Time in min	Eluent A in%	Eluent B in %
0.0	100	0
0.8	100	0
0.9	20	80
1.0	20	80
1.1	100	0
1.4 (Stop) ^a^	100	0

^a^ The next sample is injected approximately 10 s later, thus the total analysis takes less than 1.6 min.

## References

[1] Bartlett G.J., Porter C.T., Borkakoti N., Thornton J.M. (2002). Analysis of catalytic residues in enzyme active sites. J. Mol. Biol..

[2] Owen O.E., Kalhan S.C., Hanson R.W. (2002). The key role of anaplerosis and cataplerosis for citric acid cycle function. J. Biol. Chem..

[3] Newsholme P., Lima M.M., Procopio J., Pithon-Curi T.C., Doi S.Q., Bazotte R.B., Curi R. (2003). Glutamine and glutamate as vital metabolites. Braz. J. Med. Biol. Res..

[4] Meldrum B.S. (2000). Glutamate as a neurotransmitter in the brain: Review of physiology and pathology. J. Nutr..

[5] McEntee W.J., Crook T.H. (1993). Glutamate: Its role in learning, memory, and the aging brain. Psychopharmacology.

[6] Curtis D., Watkins J. (1960). The excitation and depression of spinal neurones by structurally related amino acids. J. Neurochem..

[7] Chen P.E., Geballe M.T., Stansfeld P.J., Johnston A.R., Yuan H., Jacob A.L., Snyder J.P., Traynelis S.F., Wyllie D.J. (2005). Structural features of the glutamate binding site in recombinant NR1/NR2A *N*-methyl-d-aspartate receptors determined by site-directed mutagenesis and molecular modeling. Mol. Pharmacol..

[8] Fonnum F. (1984). Glutamate: A neurotransmitter in mammalian brain. J. Neurochem..

[9] Fu Q., Ye Q., Zhang J., Richards J., Borchardt D., Gan J. (2017). Diclofenac in Arabidopsis cells: Rapid formation of conjugates. Environ. Pollut..

[10] Rozhon W., Wang W., Berthiller F., Mayerhofer J., Chen T., Petutschnig E., Sieberer T., Poppenberger B., Jonak C. (2014). Bikinin-like inhibitors targeting GSK3/Shaggy-like kinases: Characterisation of novel compounds and elucidation of their catabolism in planta. BMC Plant Biol..

[11] Forde B.G., Lea P.J. (2007). Glutamate in plants: Metabolism, regulation, and signalling. J. Exp. Bot..

[12] Hayat S., Hayat Q., Alyemeni M.N., Wani A.S., Pichtel J., Ahmad A. (2012). Role of proline under changing environments: A review. Plant Signal. Behav..

[13] Yaronskaya E., Vershilovskaya I., Poers Y., Alawady A.E., Averina N., Grimm B. (2006). Cytokinin effects on tetrapyrrole biosynthesis and photosynthetic activity in barley seedlings. Planta.

[14] Ninomiya K. (1998). Natural occurrence. Food Rev. Intern..

[15] Sorrequieta A., Ferraro G., Boggio S.B., Valle E.M. (2010). Free amino acid production during tomato fruit ripening: A focus on l-glutamate. Amino Acids.

[16] Friedman M. (2004). Applications of the ninhydrin reaction for analysis of amino acids, peptides, and proteins to agricultural and biomedical sciences. J. Agric. Food Chem..

[17] Koop D.R., Morgan E.T., Tarr G.E., Coon M.J. (1982). Purification and characterization of a unique isozyme of cytochrome P-450 from liver microsomes of ethanol-treated rabbits. J. Biol. Chem..

[18] Bidlingmeyer B.A., Cohen S.A., Tarvin T.L. (1984). Rapid analysis of amino acids using pre-column derivatization. J. Chromatogr..

[19] Cohen S.A., Bidlingmeyer B.A., Tarvin T.L. (1986). PITC derivatives in amino acid analysis. Nature.

[20] Edman P. (1950). Method for determination of the amino acid sequence in peptides. Acta Chem. Scand..

[21] Lindroth P., Mopper K. (1979). High performance liquid chromatographic determination of subpicomole amounts of amino acids by precolumn fluorescence derivatization with o-phthaldialdehyde. Anal. Chem..

[22] Radjai M.K., Hatch R.T. (1980). Fast determination of free amino acids by ion-pair high-performance liquid chromatography using on-line post-column derivatization. J. Chromatogr. A.

[23] Roth M. (1971). Fluorescence reaction for amino acids. Anal. Chem..

[24] Altmann F. (1992). Determination of amino sugars and amino acids in glycoconjugates using precolumn derivatization with o-phthalaldehyde. Anal. Biochem..

[25] Molnár-Perl I. (2011). Advancement in the derivatizations of the amino groups with the o-phthaldehyde-thiol and with the 9-fluorenylmethyloxycarbonyl chloride reagents. J. Chromatogr. B.

[26] Cohen S.A., Michaud D. (1993). Synthesis of a fluorescent derivatizing reagent, 6-aminoquinolyl-*N*-hydroxysuccinimidyl carbamate, and its application for the analysis of hydrolysate amino acids via high-performance liquid chromatography. Anal. Biochem..

[27] Dahl-Lassen R., van Hecke J., Jørgensen H., Bukh C., Andersen B., Schjoerring J.K. (2018). High-throughput analysis of amino acids in plant materials by single quadrupole mass spectrometry. Plant Methods.

[28] Salazar C., Armenta J.M., Shulaev V. (2012). An UPLC-ESI-MS/MS assay using 6-aminoquinolyl-*N*-hydroxysuccinimidyl carbamate derivatization for targeted amino acid analysis: Application to screening of *Arabidopsis thaliana* mutants. Metabolites.

[29] Piraud M., Vianey-Saban C., Petritis K., Elfakir C., Steghens J.P., Bouchu D. (2005). Ion-pairing reversed-phase liquid chromatography/electrospray ionization mass spectrometric analysis of 76 underivatized amino acids of biological interest: A new tool for the diagnosis of inherited disorders of amino acid metabolism. Rapid Commun. Mass Specrom..

[30] Nemkov T., D’Alessandro A., Hansen K.C. (2015). Three-minute method for amino acid analysis by UHPLC and high-resolution quadrupole orbitrap mass spectrometry. Amino Acids.

[31] Annesley T.M. (2003). Ion suppression in mass spectrometry. Clin. Chem..

[32] Boggio S.B., Palatnik J.F., Heldt H.W., Valle E.M. (2000). Changes in amino acid composition and nitrogen metabolizing enzymes in ripening fruits of *Lycopersicon esculentum* Mill. Plant Sci..

[33] Fuke S., Konosu S. (1991). Taste-active components in some foods: A review of Japanese research. Physiol. Behav..

[34] Polta J.A., Johnson D.C. (1983). The direct electrochemical detection of amino acids at a platinum electrode in an alkaline chromatographic effluent. J. Liq. Chromatogr..

[35] Clarke A.P., Jandik P., Rocklin R.D., Liu Y., Avdalovic N. (1999). An integrated amperometry waveform for the direct, sensitive detection of amino acids and amino sugars following anion-exchange chromatography. Anal. Chem..

[36] Welch L.E., LaCourse W.R., Mead D.A., Johnson D.C., Hu T. (1989). Comparison of pulsed coulometric detection and potential-sweep-pulsed coulometric detection for underivatized amino acids in liquid chromatography. Anal. Chem..

[37] Sherwood R.A. (1990). Amino acid measurement by high-performance liquid chromatography using electrochemical detection. J. Neurosci. Methods.

[38] Sierra T., Crevillen A.G., Escarpa A. (2017). Derivatization agents for electrochemical detection in amino acid, peptide and protein separations: The hidden electrochemistry?. Electrophoresis.

[39] Pérez-de la Mora M., Méndez-Franco J., Salceda R., Riesgo-Escovar J.R. (1989). A glutamate dehydrogenase-based method for the assay of L-glutamic acid: Formation of pyridine nucleotide fluorescent derivatives. Anal. Biochem..

[40] Bernt E., Bergmeyer H.U., Bergmeyer H.U. (1974). L-Glutamate UV-assay with gutamate dehydrogenase and NAD. Methods of Enzymatic Analysis.

[41] Sowerby J.M., Ottaway J.H. (1966). The enzymic estimation of glutamate and glutamine. Biochem. J..

[42] Graham L.T., Aprison M.H. (1966). Fluorometric determination of aspartate, glutamate, and gamma-aminobutyrate in nerve tissue using enzymic methods. Anal. Biochem..

[43] Mutaguchi Y., Ohmori T., Sakuraba H., Yoneda K., Doi K., Ohshima T. (2011). Visible wavelength spectrophotometric assays of l-aspartate and d-aspartate using hyperthermophilic enzyme systems. Anal. Biochem..

[44] Sanger F. (1945). The free amino groups of insulin. Biochem. J..

[45] Morton R.C., Gerber G.E. (1988). Amino acid analysis by dinitrophenylation and reverse-phase high-pressure liquid chromatography. Anal. Biochem..

[46] Lateef M., Siddiqui K., Saleem M., Iqbal L. (2012). Estimation of monosodium glutamate by modified HPLC method in various Pakistani spices formula. J. Chem. Soc. Pak..

[47] Anthon G.E., LeStrange M., Barrett D.M. (2011). Changes in pH, acids, sugars and other quality parameters during extended vine holding of ripe processing tomatoes. J. Sci. Food Agric..

[48] Salunkhe D.K., Jadhav S.J., Yu M.H. (1974). Quality and nutritional composition of tomato fruit as influenced by certain biochemical and physiological changes. Qual. Plant.

[49] Sorrequieta A., Abriata L.A., Boggio S.B., Valle E.M. (2013). Off-the-vine ripening of tomato fruit causes alteration in the primary metabolite composition. Metabolites.

[50] Agius C., von Tucher S., Poppenberger B., Rozhon W. (2018). Quantification of sugars and organic acids in tomato fruits. MethodsX.

[51] Van Deemter J.J., Zuiderweg F.J., Klinkenberg A. (1956). Longitudinal diffusion and resistance to mass transfer as causes of nonideality in chromatography. Chem. Eng. Sci..

